# Biomarkers of Amyotrophic Lateral Sclerosis: Current Status and Interest of Oxysterols and Phytosterols

**DOI:** 10.3389/fnmol.2018.00012

**Published:** 2018-01-31

**Authors:** Anne Vejux, Amira Namsi, Thomas Nury, Thibault Moreau, Gérard Lizard

**Affiliations:** ^1^Team Biochemistry of the Peroxisome, Inflammation and Lipid Metabolism EA 7270, INSERM, University of Bourgogne Franche-Comté, Dijon, France; ^2^Laboratoire de Neurophysiologie Fonctionnelle et Pathologies, UR11ES/09, Faculté des Sciences Mathématiques, Physiques et Naturelles de Tunis, Université de Tunis El Manar – Bienvenue, Tunis, Tunisia; ^3^Department of Neurology, University Hospital/University Bourgogne Franche-Comté, Dijon, France

**Keywords:** oxysterols, phytosterols, lipids, neurodegenerative disease, amyotrophic lateral sclerosis, biomarker, LXR signaling, oxidative stress

## Abstract

Amyotrophic lateral sclerosis (ALS) is a non-demyelinating neurodegenerative disease in adults with motor disorders. Two forms exist: a sporadic form (90% of cases) and a family form due to mutations in more than 20 genes including the Superoxide dismutase 1, TAR DNA Binding Protein, Fused in Sarcoma, chromosome 9 open reading frame 72 and VAPB genes. The mechanisms associated with this pathology are beginning to be known: oxidative stress, glutamate excitotoxicity, protein aggregation, reticulum endoplasmic stress, neuroinflammation, alteration of RNA metabolism. In various neurodegenerative diseases, such as Alzheimer’s disease or multiple sclerosis, the involvement of lipids is increasingly suggested based on lipid metabolism modifications. With regard to ALS, research has also focused on the possible involvement of lipids. Lipid involvement was suggested for clinical arguments where changes in cholesterol and LDL/HDL levels were reported with, however, differences in positivity between studies. Since lipids are involved in the membrane structure and certain signaling pathways, it may be considered to look for oxysterols, mainly 25-hydroxycholesterol and its metabolites involved in immune response, or phytosterols to find suitable biomarkers for this pathology.

## Introduction

Neurodegenerative diseases represent a major public health issue since many of them affect all age groups. Neurodegenerative diseases are classified into two subfamilies: demyelinating neurodegenerative diseases [such as multiple sclerosis (MS), peroxisomal leukodystrophies (X-ALD)] and non-demyelinating neurodegenerative diseases [including Alzheimer’s disease (AD), Parkinson’s disease (PD), Huntington’s disease, Niemann–Pick disease, amyotrophic lateral sclerosis (ALS)]. These diseases are characterized by disorders of brain functions involving one or more of these mechanisms: oxidative stress, inflammation and cell death. An American study, cross-checking the results of ten studies, showed that there were 223,000 people affected by ALS worldwide but that this figure would increase by 69% in 2040, mainly due to the aging of the population. These studies underline the fact that there is a need to increase knowledge about this disease, such as the identification of early biomarkers of the disease or patient follow-up, which will improve patient care.

## General Features of Amyotrophic Lateral Sclerosis and Current Treatments

Amyotrophic lateral sclerosis was first described in 1869 by Dr. Jean-Martin Charcot. In France, this pathology quickly became known as “Charcot’s disease.” ALS is a neurodegenerative disease, affecting adults, characterized mainly by selective and progressive degeneration of corticosteroid motor neurons and spinal and bulbar motor neurons. The loss of activity of corticosteroid-spinal neurons leads to hyper-reflexia, reversal of Babinski’s sign and spasticity, that of spinal and bulbar motor neurons leads to progressive muscle weakness, hyporeflexia, cramps and fasciculations. ALS is often associated with frontotemporal dementia (DFT) characterized by behavioral disorders.

Depending on the level of ALS, whether higher or lower, two types of ALS are described: bulbar and spinal ALS. The bulbar ALS will affect the orofacial region, while spinal ALS will initially affect the individual’s limbs resulting in muscle atrophy, cramps, and fasciculations. Depending on the neurons affected, the signs of the disease are not the same when it develops, but in all cases, the ultimate stage is progression from paralysis to the reach of respiratory muscles. After the onset of the first symptoms, patients survive between 3 and 5 years (Mulder et al., 1986). In order to better diagnose ALS, criteria have been developed that distinguish between suspected ALS, possible ALS, probable ALS and defined ALS.

The risk factors associated with ALS are related to genetic mutations that predispose or aggravate disease and/or lifestyles including environmental risk factors [exposure to heavy metals (Strickland et al., 1996), pesticides (Bonvicini et al., 2010; Malek et al., 2012), herbicides, solvents, traumatic factors, agricultural work, smoking (Nelson et al., 2000), alcoholism, intensive athletic activity (professional athletes) (Chio et al., 2005; Okamoto et al., 2007)].

There is a sporadic and family form. The sporadic form occurs in 90% of ALS cases. Both forms are clinically identical even if the severity is higher in the case of family forms.

Patients are managed in a multidisciplinary way, combining neurologists, pneumologists, nutritionists, dieticians, physiotherapists, etc. Few drugs are available; riluzole and vitamin E are used. Riluzole is an anti-glutamatergic agent derived from benzothiazoles, which blocks excitotoxicity related to glutamate (Bryson et al., 1996). This treatment extends the lifespan and delays artificial respiration (Bensimon et al., 1994; Lacomblez et al., 1996; Miller et al., 2007). It has no effect on other disease characteristics such as motor or muscular strength. Vitamin E (a set of eight organic molecules, four tocopherols and four tocotrienols) can also be used as a treatment for ALS by slowing the onset and progression of the disease (Gurney et al., 1996). As with cardiovascular disease, the use of vitamin E is still debated (Graf et al., 2005). However, one study found that a combination of riluzole and vitamin E was effective in slowing the progression of the disease but had no effect on survival (Desnuelle et al., 2001).

## Genetic Factors Associated with Amyotrophic Lateral Sclerosis

With regard to family/genetic forms, more than 20 genes have been identified for their involvement or association with ALS (**Table 1**). The genes identified have different functions in the cell, making it difficult to identify a common mechanism. The main ones will be quoted here: *Superoxide dismutase 1* (SOD1), *TAR DNA binding protein* (TARDBP), *fused in sarcoma* (FUS), *chromosome 9 open reading frame 72* (C9ORF72) and *vesicle-associated membrane protein-associated protein B* (*VAPB)*.

**Table 1:** Table of the main genes involved in amyotrophic lateral sclerosis (adapted from http://alsod.iop.kcl.ac.uk/).

Classification	Genes	Chromosomal localization	Proteins/functions
ALS 1	*SOD1*	21q22.11	**SOD1**: detoxification of cytosolic free radicals
ALS 2	*ALS2 Alsine*	2q33.2	**Alsine**: no known function, present on the cytosolic side of neuron endosomes
ALS 3	*ALS3*		*Unknown*
ALS 4	*SETX*	9q32.13	**Senataxine**: DNA/RNA helicase domain of little known function
ALS 5	*SPG11*	15q14	**Spatacsin**: role in the plasticity of neurites by maintaining stability of the cytoskeleton, regulation of synaptic vesicular transport
ALS 6	*FUS*	16p11.2	**FUS**: transcription, splicing regulation, RNA biogenesis, formation of stress granules
ALS 7	*ALS7*	20p13	*Unknown*
ALS 8	*VAPB*	20q13.33	**Vesicle-associated membrane protein-associated protein B (VAPB)**: vesicular transport and elimination of abnormal conformation proteins (PRU pathway); control of lipid metabolism and lipid transfer between endoplasmic reticulum and organelles
ALS 9	*ANG*	14q11.1	**Angiogenine**: angiogenic actor and trophic factor for motor neurons
ALS 10	*TARDBP*	1p36.22	**TAR DNA binding protein (TDP-43)**: transcription, splicing, transport of mRNAs
ALS 11	*FIG 4*	6q21	**Polyphosphoinositide phosphatase:** regulation of the cell concentration of PI (3,5)P2 which modulates the retrograde transport of endoplasmic vesicles to the golgi
ALS 12	*OPTN*	10p13	**Optineurine**: cell morphogenesis, membrane transport, vesicular, activation of transcription
ALS 13	*ATXN2*	12q23-q24.1	**Ataxine-2**: interaction with TDP-43
ALS 14	*VCP*	9p13	**Valosin-containing protein (VCP)**: vesicular transport of ATP
ALS 15	*UBQLN2*	Xp11.21	**Ubiquiline 2**: protein degradation
ALS 16	*SIGMAR1*	9p13	**Sigma non-opioid intracellular receptor 1 (SIGMAR1)**: neuroprotective membrane receptor
ALS 17	*CHMP2B*	3p12.1	**Charged multivesicular body protein 2B**: formation of multivesicular bodies (MVB) and exit of endosomal cargo proteins into MVBs
ALS 18	*PFN1*	17p13.3	**Profiline 1**: conversion of monomeric actin- (G) to filamentous actin-(F)
ALS 19	*ERBB4*	2q33.3-q34	**Receptor tyrosine-protein kinase erbB-4**: receptor for neuregulins, and members of the EGF family, regulation of central nervous system development, transcription, cell proliferation, differentiation, migration, and apoptosis
ALS 20	*HNRNPA1*	12q13.1	**Heterogeneous nuclear ribonucleoprotein A1**: transport of mRNAs from nucleus to cytoplasm, modulation of splicing
ALS 21	*MATR3*	5q31.2	**Matrin 3**: transcription, nuclear retention of defective RNAs, regulation of innate immune system response mediated by DNA viruses
ALS-FTD2	*CHCHD10*	22q11.23	**Coiled-coil-helix-coiled-coil-helix domain-containing protein 10**: maintains mitochondrial organization and structure of mitochondrial ridges
ALS-FTD1	*C9ORF72*	9p21	**Guanine nucleotide exchange C9orf72**: RNA binding, autophagy regulation
ALS	*DCTN1*	2p13	**Dynactine**: association with dynein providing axonal retrograde transport
ALS	*SQSTM1*	5q35	**Sequestosome 1/p62**: autophagy (autophagic formation and degradation of cytoplasmic inclusions containing ubiquitinylated proteins)
ALS	*UNC13A*	19p13.12	**Unc-13 homolog A**: vesicle maturation during exocytosis, involvement in neurotransmitter release, synaptic vesicle maturation
ALS	*DAO*	12q24	**D-amino-acid oxidase**: regulation of D-serine neuromodulator levels in the brain, contribution to dopamine synthesis, elimination of D-amino acids accumulated during aging
ALS	*NEFH*	22q12.1-q13.1	**Neurofilament heavy polypeptide**: maintaining the neural caliber
ALS	*PRPH*	12q12	**Peripherin**: class III intermediate filament protein
ALS	*TAF15*	17q11.1-q11.2	**TATA-binding protein-associated factor 2N**: initiation of transcription
ALS	*SPAST*	2p24	**Spastine**: recognition and cutting of polyglutamylated microtubules
ALS	*ELP3*	8p21.1	**Elongator complex protein 3**: involvement in transcriptional elongation
ALS	*LMNB1*	5q23.2	**Lamin B1**: component of the nuclear lamina, provides a framework for nuclear envelope and possible interaction with chromatin

*For each gene, the classification, chromosomal location and functions of the associated protein are indicated.*

The gene coding for the cytosolic copper-zinc superoxide dismutase protein superoxide dismutase, which is involved in cellular detoxification and protection against free radicals, is one of the genes involved in ALS (Rosen et al., 1993). There are more than 170 mutations targeting the 5 exons of this gene, all of which are predominantly transmitted with the exception of the D90A mutation and do not all involve the same phenotype^1^ (Andersen et al., 2003; Turner and Talbot, 2008; Tortelli et al., 2013; Renton et al., 2014). The evidence of the involvement of this gene has led to the establishment of an animal model for studying the disease, which has shown the involvement of different mechanisms that can participate in the degeneration of motor neurons such as oxidative stress, protein aggregates, mitochondrial defects, and glutamate excitotoxicity (Gurney, 1994; Ripps et al., 1995).

The other gene involved is the *TDP-43* gene which codes for a protein that binds to DNA and RNA and thus participates in the transcription, splicing of RNA (Buratti and Baralle, 2001; Wang et al., 2004; Neumann et al., 2006). The “false meaning” mutations that affect this gene lead to the presence of ubiquitinylated TDP-43 inclusions in the neuron cytoplasm leading to their death. Mouse models with the mutant human TDP-43 protein have been developed but have a very short lifespan (14–49 days) (Stallings et al., 2010) Mouse models expressing the human WT protein have the same characteristics, making it difficult to use the human protein in a mouse model.

The *FUS* gene also encodes a protein binding to RNA and DNA involved in transcription, splicing regulation, RNA biogenesis, and the formation of stress granules that could be responsible for neuron death (Deng et al., 2014). As with the TDP-43 protein, the mouse model expressing the human FUS protein has a short lifespan and a very aggressive phenotype (Mitchell et al., 2013).

Recently, the *C9ORF72* gene has been described as implicated in ALS by the discovery of a repetition of GGGGCCG expansion in a non-coding region of this gene. Three mechanisms could be used to explain its involvement: sequestration of RNA-binding proteins, formation of dipeptides (because of repetition), and haplo-insufficiency (Koppers et al., 2015).

More recently, mutations have been identified in the *VAPB* gene (Nishimura et al., 2004; Chen et al., 2010). The VAPB protein is involved in the vesicular transport and elimination of abnormal proteins (UPR) but also in lipid metabolism and their transfer from the endoplasmic reticulum to the organelles. The mutated form VAPB-P56S, discovered in a Brazilian family, would bind to the wild protein and prevent the wild protein from inducing the UPR pathway (Suzuki et al., 2009). Protein aggregates are also observed leading to the death of motor neurons. In the spinal cord of ALS patients, he also showed a decrease in expression of the VAPB protein (Anagnostou et al., 2010).

Other genes are involved such as *CHMP2B*, and *OPTN* (vesicular traffickers), *DCTN1* (axonal transport), *ANG* (angiogenesis), *ATXN2* (translation RNA), *UBQLN2* (proteasome), *PFN1* (cytoskeleton), and *SQSTM1* (autophagy) (**Table 1**).

## Cell Dysfunctions and Molecular Mechanisms Associated with Amyotrophic Lateral Sclerosis

The identification of the genes involved in ALS has identified some of the causes of the disease even though the mechanism of the disease remains poorly understood. The identification of mutations in the *SOD1* gene has made it possible to involve oxidative stress, in fact SOD1 is one of the main antioxidant defense used to fight against the accumulation of free radicals. Several hypotheses have been put forward regarding toxicity due to these mutations: either a loss of SOD1 dismutase function leading to superoxide overproduction (O_2_^⋅-^) (Beckman and Crow, 1993; Deng et al., 1993) or inactivation of SOD not mutated by the mutated form of SOD1 (Rosen et al., 1993), or an increase in SOD1 activity leading to an increase in oxidative stress. Another important mechanism is glutamate excitotoxicity. This process is caused by excessive stimulation of the glutamatergic receptors NMDA (*N*-methyl-D-aspartic acid) and AMPA (α-amino-3-hydroxy-5-methyl-4-isoxazolepropionic acid) in the post-synaptic neurons. Indeed, glutamate is synthesized at the presynaptic terminal, diffused through the synaptic space and activates receptors present at the post-synaptic neuron (AMPA and NMDA), triggering a potential for action activating voltage-dependent calcium channels, followed by a calcium input into the cell. Because glutamate is a very powerful neurotransmitter, it is important that its action be short-lived. The glial cells, mainly astrocytes (via GLT-1 and EAAT2 glutamate transporters), recapture glutamate, transform it into glutamine which is released, to be taken up by nerve cells which will transform it into glutamate for future use as a neurotransmitter (Bonafede and Mariotti, 2017; Murphy-Royal et al., 2017). In patients with ALS, plasma levels have been shown to be much higher than in healthy subjects, correlated with the duration of the disease, but also with abnormalities in glutamate recapture and expression of glutamate transporters (EAAT2 transporter) (Rothstein et al., 1992; Plaitakis and Constantakakis, 1993; Ferrarese et al., 2001). This mechanism leads to neuronal toxicity and cell death. Similar to what is observed in AD and PD; there are cytoplasmic inclusions (ubiquitinylated and granular eosinophilic inclusions) and protein aggregates in motor neurons of ALS patients (Xiao et al., 2006). Protein aggregates can be composed of different proteins: SOD1 [present only in family forms of ALS (Rosen et al., 1993; Blokhuis et al., 2013); TDP-43 (Arai et al., 2006), FUS (Neumann et al., 2009b; Doi et al., 2010), optineurine, ataxin-2, and ubiquiline-2 (Blokhuis et al., 2013)]. These aggregates are involved in neuronal apoptosis. It is not very clear, however, whether they are the cause, consequence or a defense. Ubiquitinylated aggregates of TDP-43 are present in spinal and cortical neurons, hippocampus, and glial cells in patients with sporadic ALS and in patients with non-family forms of SOD1 (Mackenzie et al., 2007; Tan et al., 2007). Optineurin aggregates are present only in Japanese populations. FUS protein aggregates occur very early and play a central role in the disease pathogenesis (Neumann et al., 2009b). ALS is also characterized by alterations in the structure and functioning of mitochondria: vacuolization and swelling of mitochondria with decrease in the activity of complex I of the respiratory chain (Mattiazzi et al., 2002). Reticulum stress, neuroinflammation and alteration of RNA metabolism are also part of the pathophysiological mechanisms. Reticulum stress is thought to be induced by morphological changes in the reticulum but also by the accumulation of abnormal proteins (Nishitoh et al., 2008; Oyanagi et al., 2008). Indeed, in ALS patients and in the SOD1 mutated animal model, morphological abnormalities of the endoplasmic reticulum have been shown: rough endoplasmic reticulum, dilated with detachment of ribosomes in mice and a rough and fragmented reticulum in patients (Oyanagi et al., 2008). The mutated protein SOD1 also accumulates in the endoplasmic reticulum, forming aggregates and interacting with chaperone proteins such as Derlin-1. This leads to an accumulation of abnormal proteins in the reticulum endoplasmic and leads to stress (Nishitoh et al., 2008). Neuroinflammation, on the other hand, would involve astrocytes and microglia. In patients or animal models, astrocytosis is present as well as activation of the microglia characterized by an alteration of the transition to the M2 state. Early in the disease, the microglia have an M2 phenotype [production of anti-inflammatory cytokines (Interleukin-3, Interleukin-4, Transforming Growth Factor β), neurotrophic factors] that promotes neuronal repair and regeneration. During the pathology, microglial cells acquire an M1 phenotype: secretion of ROS, pro-inflammatory cytokines [(Tumor Necrosis Factor α, Interleukin 1β, Interleukin-6, Interleukin-23, Interleukin-12, Interleukin-8), and neurotoxic molecules that promote motor neuron death (Almer et al., 1999; Elliott, 2001)]. During the progression of the disease, astrogliosis is also involved, leading to negative regulation of the EAAT2 transporter contributing to glutamate excitotoxicity (Howland et al., 2002). A study also showed that astrocytes in ALS patients developed positive regulation of genes encoding chemokines, proinflammatory cytokines, and components of the complement cascade, compounds that can exacerbate damage to neurons (Zhao et al., 2013). Concerning RNA metabolism, mutations in the *TDP-43* and *FUS* genes lead to the formation of stress granules in the cytoplasm, which would then be toxic to neurons and disturbance of the splicing phenomenon (Ferraiuolo et al., 2011). The pathophysiological mechanisms involved in ALS are therefore very diverse, which may explain the limited treatment available and overall patient management.

## Lipids Involvement in Amyotrophic Lateral Sclerosis

### Clinical Arguments

Amyotrophic lateral sclerosis has been described as having energy metabolism disorders. Among the lipids that might be involved, cholesterol and its forms of transport were naturally studied. Cholesterol has two origins, it is either ingested in the food bowl and absorbed in the intestine, or neosynthesized by hepatocytes and peripheral cells. The liver is the main organ of cholesterol breakdown. Cholesterol is synthesized in the cytoplasm of cells (especially the intestine and liver) from hydroxy-methylglutaryl-CoA (HMG-CoA). HMG-CoA is derived from the condensation of 3 acetyl-CoA from peroxisomes (Doria et al., 2016; Zarrouk et al., 2017) Short chain fatty acids (C8) and leucine are also good substrates for cholesterol synthesis. Cholesterol is not water-soluble and is therefore insoluble in biological media. As a result, the transport of lipids in plasma and lymph is ensured by water-soluble macromolecular complexes composed of various lipids and proteins: lipoproteins. There are several classes of lipoproteins: chylomicrons (CM), very low density lipoproteins (VLDL: Very Low Density Lipoprotein), lipoproteins of intermediate density (IDL: Intermediary Density Lipoprotein), low density lipoproteins (LDL: Low Density Lipoprotein), high density lipoproteins (HDL: High Density Lipoprotein). The transport of dietary lipids from the intestine to the liver is carried out by CM (exogenous enterohepatic route), then from the liver to peripheral tissues by VLDL, IDL, and LDL. The return route of lipids is ensured by HDLs, which return lipids from peripheral tissues to the liver, thus eliminating cholesterol in native form or after transformation into bile acids. In the case of hyperlipoproteinemia, total cholesterol, HDL and LDL are measured via a blood test to assess cholesterol metabolism, HDL being considered “good cholesterol” and LDL “bad cholesterol.” It was therefore natural to measure the levels of total cholesterol, triglycerides, LDL and HDL in blood tests. Various studies have followed one another to try to correlate the lipid profile with the appearance or follow-up of the pathology in humans or in mouse models. A Swedish study followed patients for 20 years and showed that high LDL levels and a high LDL/HDL ratio were associated with a higher risk of developing ALS (Mariosa et al., 2017). The authors proposed that alterations in lipid metabolism could be used as prodromal symptoms decades before ALS diagnosis (Mariosa et al., 2017). The difference between men and women is also highlighted in the results observed. This may explain the differences between studies. Dupuis et al. (2008) also showed a positive association between total cholesterol levels, LDL, LDL, and LDL/HDL and ALS with higher levels of LDL and LDL/HDL ratio among ALS patients (369 patients) than in controls (286 healthy subjects). This is not the case with HDL and triglyceride levels. In another study of serum in 92 ALS patients at diagnosis compared to 92 healthy subjects with the same age, sex, and BMI characteristics, there was a positive correlation for total cholesterol, triglyceride, and LDL levels in women but no association in men for HDL, total cholesterol, LDL, and triglyceride (Ikeda et al., 2012). Conversely, Yang et al. (2013) show that there is no correlation in women but a negative association in men. This same observation is found in mice (Kim et al., 2011). In a study in south-west China, total cholesterol, triglyceride, LDL or LDL/HDL levels are on average the same between patients and controls, while ALS patients with higher triglyceride levels have a longer survival rate (Huang et al., 2015). This observation was also validated by Dorst et al. (2011), who showed that elevated triglyceride and cholesterol levels correlated with increased life expectancy and suggested that lipid metabolism and nutritional status of ALS patients were important prognostic factors. The serum and CSF of 35 ALS patients versus 24 controls were analyzed in terms of cholesterol and intermediates of the bile acid synthesis pathway. In ALS patients, cholesterol levels are high in CSF. On the other hand, intermediates in the bile acid synthesis pathway are reduced in ALS patients compared to control, indicating that the pathway for removing excess cholesterol is no longer operational in the central nervous system and may lead to neuronal cell toxicity (Abdel-Khalik et al., 2017). Recently, Delaye et al. (2017) have studied the serum of 30 ALS patients compared to 30 controls by analyzing total cholesterol levels, HDL and LDL. ALS patients have higher total cholesterol and HDL and LDL levels than controls, but LDL/HDL ratios do not differ between the two groups and there is no association with disease progression. They also showed that there was no difference in lipid profile between the 15 ALS patients treated with tocopherol and the 15 untreated ALS patients (Delaye et al., 2017). Thus, there are strong differences between studies based on parameters related to energy metabolism of lipids that are for example sex and/or age related. It may therefore be interesting to turn to the other functions of lipids that may be their involvement in membrane structure and fluidity or in signaling pathways.

### Physiopathological Arguments: Lipids, Membrane Structure, and Cell Signaling

In ALS, the energetic metabolism and in particular that of the muscle is strongly disturbed, which has led to an interest in lipids, because of their involvement in energy metabolism but also because of their structural roles in cell membranes and cell signaling.

We will focus here more on the structural and signaling role attributed to lipids.

Lipids are involved at the structural level in membrane fluidity, synapse stability and signal transmission optimization. Modulations at the level of these different aspects are involved in ALS. However, very little study explores the relationship between lipids and ALS at the membrane level despite changes in lipid levels observed in ALS. In the spinal cord of SOD1m mice and ALS patients, increases in sphingolipids, cholesterol and lipid peroxidation have been shown (Cutler et al., 2002). A decrease in membrane fluidity was also demonstrated in the spinal cord of SOD1m mice, induced by a decrease in the proportion of polyunsaturated fatty acids (PUFAs) [e.g., docosahexaenoic acid (DHA) (C22:6 *n*-3)] and leading to a decrease in membrane fluidity (Ilieva et al., 2007). This loss of membrane fluidity is also due to oxidative stress and lipid peroxidation (Miana-Mena et al., 2011). The composition of the membrane in cholesterol and sphingolipids such as sphingomyelin can also lead to changes in lipid rafts that are involved in certain signal transduction pathways. It has been shown that motor neurons isolated from 15-day-old rats can undergo excitotoxicity in association with receptor tyrosine kinase B (TrkB) activation induced by the interaction between brain-derived neurotrophic factor (BDNF) and its receptor (Fryer et al., 2000; Hu and Kalb, 2003). Other studies have shown that excitotoxicity can be inhibited by inhibiting the effects of BDNF (Mojsilovic-Petrovic et al., 2006). It was then shown that TrkB, the adenosine A2a G-protein-coupled receptor, and src-family kinases can be present in lipid rafts and non-lipid raft regions (Mojsilovic-Petrovic et al., 2006). If lipid rafts, such as methyl-β-cyclodextrin, are broken off with lipid rafts, there is protection against BDNF induced excitotoxicity (Mojsilovic-Petrovic et al., 2006). The importance of membrane lipids in ALS is also supported by the presence of increased BDNF in the muscle of ALS patients and expression by motor neuropathic patients with anti-ganglioside antibodies (Pestronk and Choksi, 1997; Kust et al., 2002; Mizutani et al., 2003).

Lipids can also play a role in cellular signaling as a second messenger or by modulating exchanges. PUFAs, whose rates are changed during ALS (Ilieva et al., 2007), can act at the cellular signaling level since they are able to bind to transcription factors/nuclear receptors such as liver-X receptor (LXR) and retinoic-X receptor (RXR) (Yoshikawa et al., 2002). LXR α and β are inductive transcription factors for which oxysterols are the natural ligands. LXRs form heterodimers with RXR that will bind to LXR-response elements (LXRs) at the level of the target gene promoter. These increase the expression of genes involved in energy homeostasis that can be deregulated in ALS. Among PUFA, eicosapentaenoic acid (EPA; C20:5 *n*-3), DHA, arachidonic acid (ARA; C20:4 *n*-6) are capable of interacting with these receptors. PUFAs have been shown to inhibit binding of heterodimer LXR/RXR to LXRE (Yoshikawa et al., 2002). Using an oil enriched with EPA, it has been shown that PUFA can modulate the expression of certain genes by acting via receptors LXR and RXR (Gillies et al., 2012). The involvement of these receptors has also been shown in the use of transgenic mice LXRβ which possess an impairment of motor performance from the age of 7 months and progresses to hind limb paralysis (Andersson et al., 2005). A study of *LXR* gene polymorphism and its influence on ALS progression was conducted in 438 ALS patients versus 330 healthy controls. An interaction between the LXRα and LXRβ genes and the phenotype and the risk of developing ALS is demonstrated. Indeed, the association between SNP genotypes of the LXRα gene and the late age of onset was validated by this study, as was the fact that the C/C of SNP genotype rs2695121 from the LXRβ gene is associated with a 30% increase in ALS survival (Mouzat et al., 2017).

Polyunsaturated fatty acids can also be considered active molecules that may have neuroprotective, anti-inflammatory, and pro-inflammatory effects. DHA can be oxidized and given neuroprotectin D1, which promotes cell survival under stress (Bazan et al., 2011). EPA and ARA, on the other hand, can lead to the synthesis of prostaglandins and leukotrienes that are involved in inflammatory phenomena.

Lipids may therefore be involved in signaling pathways involving LXR receptors, or may induce inflammation. The studies carried out on this topic have mainly focused on PUFAs. Another class of molecules derived from a lipid, particularly cholesterol and its derivatives, may be of particular interest. Indeed, some oxysterols [cholesterol oxide products (COPs)], mainly those resulting from an oxidation on the lateral chain of cholesterol, are the natural ligands of LXR. In addition, some of these oxysterols (including those resulting from an oxidation on the sterol core and on the lateral chain of cholesterol) can be involved in cell death phenomena (autophagy, apoptosis) but also in oxidative stress and inflammation induction.

## Oxysterols and Brain Functions

Oxysterols are oxidized cholesterol derivatives/COPs with 27 carbon atoms. Oxidation can take place either on the sterol core or on the side chain. Oxysterols can be produced either by auto-oxidation or enzymatically or brought from food (eggs, dried egg powder, milk powder, cheese, clarified butter, etc.) (Przygonski et al., 2000; Boselli et al., 2001). Auto-oxidation occurs when reactive or pro-oxidant species are present [ROS, ozone, Ultraviolet (UV) light, peroxide, and hydroperoxide species]; this auto-oxidation mainly occurs on the sterol nucleus, forming 7-hydroxycholesterols (α and β), 7-ketocholesterol, epoxides 5.6 (5α, 6α and 5β, 6β), and cholestantriol (Patel et al., 1996; Lercker and Rodriguez-Estrada, 2002; Iuliano, 2011; Zerbinati and Iuliano, 2017). Various enzymes are involved in the synthesis of oxysterols and belong mainly to the cytochrome family P450 (CYP) except 25-hydroxylase. 27-hydroxylase (CYP27A1), 25-hydroxylase, 24-hydroxylase (CYP46A1), 7α-hydroxylase (CYP7A1) and CYP3A4 synthesize 27-hydroxycholesterol [or (25R)26-hydroxycholesterol], 25-hydroxycholesterol, 24- hydroxycholesterol, 7α-hydroxycholesterol, and 4β-hydroxycholesterol, respectively (Griffiths et al., 2016; Mutemberezi et al., 2016).

Oxysterols are important signaling molecules, capable of modulating the activity of many transcription factors in order, among other things, to limit the excessive accumulation of cholesterol. Indeed, the presence of an oxygenated group in addition to β-hydroxy on the carbon in position 3, allows oxysterols to be perfectly inserted into the double lipid layer of the plasma membrane (Olkkonen and Hynynen, 2009). Two categories of receptors, cytosolic and nuclear, are likely to bind oxysterols. Cytosolic receptors include OxySterol Binding Proteins (OSBPs), OSBP-related Protein (ORPs), Estrogene Binding Site antibodies (AEBS), and aryl hydrocarbon receptors (AhR). We will only focus on OSBP receptors for cytosolic receptors. OSBPs are available in two forms, OSBP1 and OSBP2, the oxysterol binding region of which is located in the carboxyl-terminal region of OSBP. 25-hydroxycholesterol is the compound with the highest affinity for OSBP1 but other oxysterols such as 7-ketocholesterol. OSBPs have many regulatory effects on the metabolism of cellular cholesterol and sphingomyelin. Overexpression of OSBP in hamster ovarian cells (CHO)-K1, shows decreased biosynthesis of cholesterol esters and ACAT activity (Lagace et al., 1997), as well as increased production of sphingomyelin (Lehto and Olkkonen, 2003). In addition, OSBPs could control the availability of oxysterols for downstream receptors [sterol responsive element binding protein (SREBP) and LXR (Liver X Receptor)] that are more directly involved in controlling cholesterol homeostasis (Lehto and Olkkonen, 2003). OSBP also has the ability to increase the synthesis of sphingolipids via its ability to target endoplasmic reticulum by interacting with VAP through its FFAT domain [two phenylalanines (FF) in an Acidic Tract] and Golgi’s apparatus by interacting with Arf1 through its Pleckstrin Homology (PH) domain (Perry and Ridgway, 2006). Nuclear receptors include SREBP, LXRs, Steroidogenic Factor-1 (SF-1) and some other nuclear receptors such as steroid X receptor (SXR). For LXRs, the most effective ligands of isoform α are those oxidized on the side chain, such as 22 (R)-hydroxycholesterol, 20(S)-hydroxycholesterol, 24(S),25-epoxycholesterol, 24-hydroxycholesterol (also named cerebrosterol), 25-hydroxycholesterol, and 27-hydroxycholesterol (Janowski et al., 1999; Ory, 2004). 7-ketocholesterol, 7α-hydroxycholesterol, and 7β-hydroxycholesterol are less effective ligands of Janowski et al. (1999).

Concerning neurodegenerative diseases, oxysterols that may be of interest are the oxysterols present at the cerebral level, mainly 24S-hydroxycholesterol which is produced in the brain, and 27-hydroxycholesterol, which enters in the brain when the permeability of the blood brain barrier is enhanced. 24S-hydroxycholesterol is the only oxysterol synthesized in the brain by neuronal cells, and it is able to cross the blood–brain barrier for further degradation in the liver (Bjorkhem, 2006; Iuliano et al., 2015). This oxysterol is the form by which cholesterol is eliminated from the brain in order to maintain constant levels of cerebral cholesterol. Despite this, other oxysterols have been shown to be present at the cerebral level, such as 27-hydroxycholesterol, which is present in cerebrospinal fluid (CSF) following abnormalities in the blood–brain barrier or blood–CSF barrier (Leoni et al., 2003; Heverin et al., 2005; Iuliano et al., 2015). In smaller amounts, 7β-hydroxycholesterol, 7-ketocholesterol and 3β, 5α-dihydroxycholestan-6-one (3β, 5α -diHC-6O) are also found in the brain (Iuliano et al., 2015).

At the moment, the involvement of 24S-hydroxycholesterol and 27-hydroxycholesterol has been shown to be involved in various neurodegenerative diseases, such as MS, AD, or cognitive mild oddly. In the case of MS, high levels of 24S-hydroxycholesterol are observed during active versus remission periods, whereas in AD and cognitive mild, there is an increase in concentrations of 24S-hydroxycholesterol and 27-hydroxycholesterol (Leoni, 2009). In addition, important accumulation of 7-ketocholesterol have been described in the brain of patients with AD (Testa et al., 2016). As this oxysterol is formed by cholesterol auto-oxidation, and as oxidative stress is involved in numerous neurodegenerative diseases, including ALS, it would be of interest to determine whether the level of this oxysterol is affected in ALS patients.

## Conventional Biomarkers of Amyotrophic Lateral Sclerosis

A very long lead time is usually required to diagnose ALS and often the patient has progressed in his (or her) disease. Several clinical and molecular biomarkers are proposed to diagnose the disease.

Among clinical biomarkers, neurophysiological approaches can be used to detect motor neuron damage: study of nerve conduction (axonal degeneration), electromyography (motor neuron damage), estimation of the number of motor units, transcranial magnetic stimulation (functional integrity of neurons), or electrical impedance myography (functional integrity and muscle structure) (Inghilleri and Iacovelli, 2011; Joyce and Carter, 2013; Turner et al., 2013). Imaging techniques can also be used as biomarkers. These include monophotonic emission tomography (SPECT), Positron emission tomography (PET), functional magnetic resonance imaging (fMRI), and diffusion tensor imaging (DTI) (Ludolph et al., 1989; Takahashi et al., 1993; Turner et al., 2005, 2009, 2013; Turner, 2011). Molecular biomarkers can be detected in body fluids: CSF, urine, blood, saliva.

In CSF, markers of blood–brain barrier dysfunction can be measured: metalloproteinases-2 and -9 (MMP-2 and MMP-9) in ALS patients (Niebroj-Dobosz et al., 2010). Markers of axonal degeneration are also present in CSF such as Tau protein, heavy chain and light chain neurofilaments, the increase in which reflects damage to the corticospinal tract (Steinacker et al., 2016). The presence of TDP-43 protein (Neumann et al., 2009a), GDNF (Glial cell-line derived neutrophic factor) (Grundstrom et al., 2000) can also be measured. Oxidative stress can also be tested with the measurement of 4-hydroxynonenal (4-HNE), 3-nitrotyrosine, 8-hydroxy-2′ -deoxyguanosine (8OH2′ dG). Some markers of inflammation such as interleukin-6 and -8 (IL-6, IL-8), prostaglandin E2 (PGE2) or monocyte chemoattractive protein-1 (MCP-1) can be detected in CSF (Ganesalingam et al., 2013). Despite the range of detectable biomarkers, they do not appear to be effective enough for the early stages of the disease but are more useful for monitoring disease progression (Tarasiuk et al., 2012).

The muscle also provides information about the disease. Indeed, expression of the neuritic growth inhibitory protein Nogo-A is increased in patients with ALS (Dupuis et al., 2002). Transcriptomic studies also separate early and late stage patients (Pradat et al., 2012). However, these tests are not carried out in the first instance because the removal of muscle tissue is painful and invasive.

Blood, plasma, or serum biomarkers have been able to demonstrate an increase in tyrosine and glutamate concentrations (Ilzecka et al., 2003), proinflammatory cytokines (IL-6), which is associated with the duration of the pathology (Ono et al., 2001), anti-HMGB1 (high mobility group box1) autoantibodies (Hwang et al., 2013) Markers of oxidative stress can also be measured such as 4-HNE, 8OH2′ dG, or nitric oxide.

Despite the possible use of these different biomarkers, it is necessary to continue to look for new biomarkers of early onset of the disease or which could help to have a better follow up of the disease. In this context, research could be directed toward the metabolism of lipids, whose alterations could contribute in the development of ALS.

## Oxysterols; Potential Biomarkers of Amyotrophic Lateral Sclerosis

Some advances in understanding the pathophysiological mechanisms of ALS show that nuclear receptors such as LXR are involved. Oxysterols have part of their ability to bind these LXR receptors but also others such as OSBP supporting a potential involvement in ALS. A serum and CSF study was conducted in non-riluzole ALS patients, riluzole-treated ALS patients and healthy subjects to measure 24S-hydroxycholesterol, 27-hydroxycholesterol, oxysterols in the brain and 25-hydroxycholesterol levels (LXR and OSBP1 ligands) (Kim et al., 2017). In the untreated ALS group, the levels of 24-hydroxycholesterol and 25-hydroxycholesterol measured in CSF are higher than in the control and treatment groups (Kim et al., 2017). The levels of 27-hydroxycholesterol (CSF) and 25-hydroxycholesterol (serum) are higher in untreated ALS patients compared to controls. The severity and progression of the disease appear to be significantly associated with serum levels of 25-hydroxycholesterol (Kim et al., 2017). The different oxysterol measurements were carried out by liquid chromatography-tandem mass spectrometry analysis. In the same article, the authors also show that 25-hydroxycholesterol induces activation of the GSK1 pathway and apoptosis in a motor neuron cell line expressing stable G93A mutant of SOD1 (Kim et al., 2017). Riluzole is capable of inhibiting 25-hydroxycholesterol induced apoptosis as well as 22 (S)-hydroxycholesterol, an LXR antagonist, showing the involvement of the LXR signaling pathway in 25-hydroxycholesterol induced death (Kim et al., 2017). Using an animal mouse model with an SOD1 mutant (mSOD1-G93A mice), it has been shown that expression of mRNAs of enzymes involved in the synthesis of 25-hydroxycholesterol (25-hydroxylase and CYP3A4) is only observed in early symptomatic states of the pathology but not in late states. Based on the increased presence of TLR-4 in the spinal cord gland of ALS patients, the authors hypothesized that 25-hydroxycholesterol induces neuronal death via GSK-3/LXR pathways following a positive regulation of inflammatory signals of the glia (Kim et al., 2017). These observations on a possible involvement of 25-hydroxycholesterol are corroborated by other observations: the 25-hydroxyhydroxyase gene is increased in autopsy tissues of ALS patients (Malaspina et al., 2001), and mutations in CYP7B1 (involved in the metabolism of 25-hydroxycholesterol) cause upper-motor-neuron degenerative diseases (Tsaousidou et al., 2008). Conversely, in another study investigating the levels of 24S-hydroxycholesterol, 27-hydroxycholesterol and 25-hydrocholesterol in the plasma of ALS patients, there was no statistically significant correlation between the levels of these oxysterols and the presence of ALS, despite levels that tend to be higher in ALS patients (Wuolikainen et al., 2014). In this article, oxysterol analyses were done by isotope dilution mass spectrometry. La Marca et al. (2016) have shown that levels of 24S-hydroxycholesterol were significantly higher in controls compared to ALS patients in both plasma and CSF. On the other hand, levels of 24S-hydroxycholesterol esterified are lower in patients than in controls in CSF, which could be due to oxidative stress that limits the activity of the LCAT enzyme responsible for esterifying 24S-hydroxycholesterol (La Marca et al., 2016). Another team focused on the familial form of ALS related to a mutation in VapB and the relationships between VapB, OSBP, and endoplasmic reticulum (Moustaqim-Barrette et al., 2014). VapB interacts with OSBP via the FFAT sequence and this interaction is required for the localization of OSBP at the endoplasmic reticulum (Loewen et al., 2003; Kaiser et al., 2005). If the interaction in VapB and OSBP is defective, this leads to a disruption of endoplasmic reticulum proteostasis causing protein accumulation in the endoplasmic reticulum and stress of the endoplasmic reticulum (Moustaqim-Barrette et al., 2014). The authors also suggest that the VAPB/OSBP pathway is required for execution of endoplasmic reticulum quality control (Moustaqim-Barrette et al., 2014). OSBP with its ability to bind cholesterol and oxysterols can play a role in the distribution of these two compounds between different organelles, potentially disrupting membrane fluidity.

These various data strongly suggest a possible involvement of oxysterols in ALS and their potential use as biomarkers. Further studies are needed to assess whether blood may be sufficient to use this type of biomarker but also to assess whether esterified forms of oxysterols could be of interest.

To broaden the discussion, another class of sterol, phytosterols, which are present in plants. These molecules are structurally related to cholesterol and are mainly C28 and C29 carbon steroid alcohols (Otaegui-Arrazola et al., 2010). These phytosterols can be oxidized, and may also be involved in the diagnosis of ALS. Some of them could impact mitochondrial functions (Lizard, 2011). The main dietary phytosterols are β-sitosterol, campesterol, and stigmasterol. These compounds are also known to be LXR α and β ligands that are involved in ALS (Kaneko et al., 2003; Plat et al., 2005). Kim colleagues have shown that β-sitosterol accentuates and accelerates the degeneration that occurs in LXRβ -/- mice. The authors suggest that signaling LXRβ and LXRα is different with β-sitosterol with activation of LXRα linked to neurodegeneration and activation of LXRβ linked to neuroprotection. Indeed, when the signalization passing through LXRβ is abnormal, the use of β-sitosterol induces neuronal damage in the spinal cord and substantia nigra (Kim et al., 2008). Another interesting observation is the positive correlation between the consumption of *Cycas micronesica* (a vegetable rich in β-sitosterol) in the Guam population and the onset of ALS. In addition, in mice mutated for SOD1, phytosterols have been shown to accumulate in the spinal cord (Cutler et al., 2002). Glucoside derivatives also appear to be involved in ALS. Indeed, β-sitosterol-β-D-glucoside promotes glutamate release in cortical sections, leading to glutamate excitotoxicity (Khabazian et al., 2002). Mice fed with cycad flour (contains different glucosides sterols) develop similar cognitive and motor disorders present for ALS as well as neurodegeneration in central nervous system regions common to the affected areas during ALS (Khabazian et al., 2002; Wilson et al., 2002). Phytosterols and their derivatives also appear to be good candidates either as molecules involved in the pathophysiology of ALS or as markers of this pathology.

**FIGURE 1:**
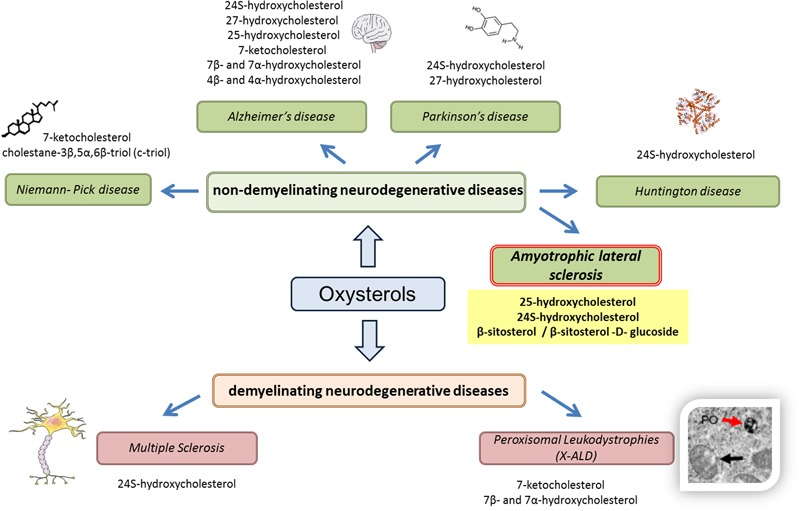
Oxysterols in various neurodegenerative diseases. Based on recent results (*in vitro, in vivo*, or clinical studies), the use of oxysterols as biomarkers is reinforced in various neurodegenerative diseases, whether demyelinating (multiple sclerosis, peroxisomal leukodystrophies) or non-demyelinating (Niemann–Pick disease, Alzheimer’s disease, Parkinson’s disease, and Huntington disease). For amyotrophic lateral sclerosis, it can also be hypothesized that oxysterols can be used as biomarkers, particularly 25-hydroxycholesterol and 24S-hydroxycholesterol, as well as phytosterols such as β-sitosterol and its glucoside derivatives.

On the basis of these recent results, some oxysterols and phytosterols may constitute SLA biomarkers, which reinforces the interest given to oxysterols as biomarkers of neurodegenerative diseases (**Figure 1**).

## Conclusion

Amyotrophic lateral sclerosis is a neurodegenerative disease involving various processes such as oxidative stress, glutamate excitotoxicity or protein accumulation, but diagnosis with early biomarkers is difficult. Given the demonstrated involvement of lipids in this pathology and the signaling pathways involved, a working hypothesis could be the use of oxysterols (especially oxysterols associated with inflammation and oxidative stress) or phytosterols as biomarkers.

## Author Contributions

AV and GL wrote the manuscript. AN (Ph.D. student), TN (Ph.D. student) and TM provided expert advice on ALS and oxysterols.

## Conflict of Interest Statement

The authors declare that the research was conducted in the absence of any commercial or financial relationships that could be construed as a potential conflict of interest.

## Footnotes

1 http://www.alsod.org
